# An NMR Study of the Bortezomib Degradation under Clinical Use Conditions

**DOI:** 10.1155/2009/704928

**Published:** 2009-04-14

**Authors:** Adele Bolognese, Anna Esposito, Michele Manfra, Lucio Catalano, Fara Petruzziello, Maria Carmen Martorelli, Raffaella Pagliuca, Vittoria Mazzarelli, Maria Ottiero, Melania Scalfaro, Bruno Rotoli

**Affiliations:** ^1^Dipartimento di Chimica Organica e Biochimica, Università Degli Studi di Napoli Federico II, Via Cynthia 6, Monte Sant'Angelo, 80126 Napoli, Italy; ^2^Dipartimento di Biochimica e Biotecnologie Mediche, Divisione di Ematologia, Università Degli Studi di Napoli Federico II, 80131 Napoli, Italy; ^3^Farmacia Centralizzata, Azienda Ospedaliera, Universitaria “Federico II”, 80131 Napoli, Italy

## Abstract

The (R)-3-methyl-1-((S)-3-phenyl-2-(pyrazine-2-carboxamido)propanamido)butyl-boronic acid, bortezomib (**BTZ**), which binds the 20S proteasome subunit and causes a large inhibition of its activity, is a peptidomimetic boronic drug mainly used for the treatment of multiple myeloma. Commercial **BTZ**, stabilized as mannitol derivative, has been investigated under the common conditions of the clinical use because it is suspected to be easily degradable in the region of its boronic moiety. Commercial **BTZ** samples, reconstituted according to the reported commercial instructions and stored at 4°C, were analyzed by high-field nuclear magnetic resonance spectroscopy in comparison with identical samples bubbled with air and argon, respectively. All the samples remained unchanged for a week. After a month, the air filled samples showed the presence of two main degradation products (6% of starting material), the N-(1-(1-hydroxy-3-methylbutylamino)-1-oxo-3-phenylpropan-2-yl) pyrazine-2-carboxamide (**BTZ1**; 5%, determined from NMR integration) and the (S)-N-(1-(3-methylbutanamido)-1-oxo-3-phenylpropan-2-yl)pyrazine-2-carboxamide (**BTZ2**; 1%, determined from NMR integration), identified on the basis of their chemical and spectroscopic properties. The **BTZ1** and **BTZ2** finding suggests that, under the common condition of use and at 4°C, commercial BTZ-mannitol is stable for a week, and that, in time, it undergoes slow oxidative deboronation which partially inactivates the product. Low temperature and scarce contact with air decrease the degradation process.

## 1. Introduction

**Figure 1:**
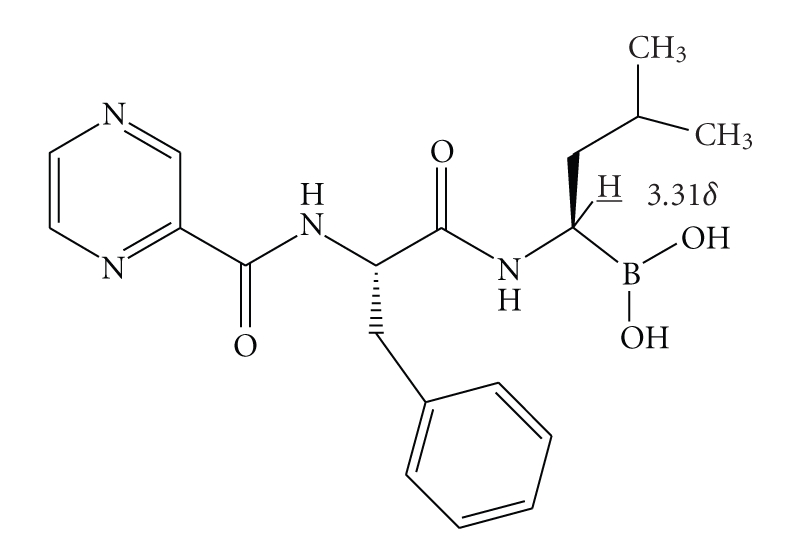
(R)-3-methyl-1-((S)-3-phenyl-2-(pyrazine-2- carboxamido)-propanamido)butylboronic acid.

Bortezomib (**BTZ**) [1], the (R)-3-methyl-1-((S)-3-phenyl-2-(pyrazine-2-carboxamido) propanamido)butylboronic acid, (**BTZ**, Figure 1), is one of the most important members of a new class of drugs, containing a boronic acid moiety, effective on a wide group of tumors. At present, it is mainly used for the treatment of multiple myeloma, a plasma cell tumor which accounts for 10% of all blood system malignancies [2, 3]. **BTZ** is a peptidomimetic compound, constituted by a modified leucine-phenylalaninedipeptide, containing a boronic acid at the C-terminal. It is able to interact with proteasome, an intracellular apparatus which brakes down damaged or unneeded proteins, inhibiting the proteolysis action [4, 5].

**Figure 2:**
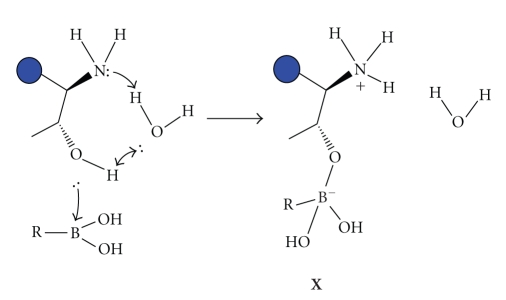
Formation of tetrahedric complex between the hydroxyl group of threonine present on the 20S *β*5 subunit of the proteasome and the boronic residue of **BTZ**. A water molecule is involved in the mechanism of complex formation [6–8].

As a boronic acid [6], **BTZ** shows high affinity for hard oxygen-containing nucleophiles according to the Lewis hard-soft acid-base theory. Specifically, peptide boronates are well-known inhibitors of serine proteases, forming a serine-boronate tetrahedral transition state complex. **BTZ**, active at subnanomolar concentrations (Ki 0.6 nM), seems to interact with the hydroxyl of a threonine present in the active site of the N-terminal of the 20S *β*5 subunit of the proteasome. Formation of a tetrahedral complex (**X**) inhibits the chymotryptic proteolytic activity, totally hampering proteasomal functions (Figure 2).

**Figure 3:**
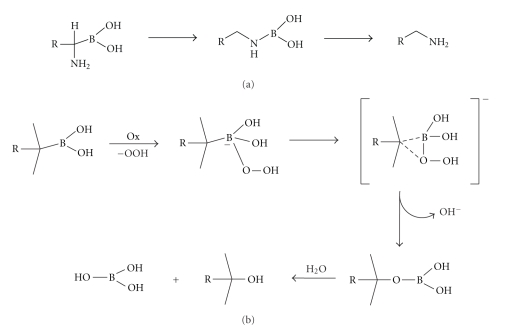
Two possible degradation processes of **BTZ** in its pharmaceutical solution form (a) degradation pathway of boronic amino acids in presence of water or other nucleophilic agents; (b) oxidative degradation pathway of boronic acids.

Generally, boronic acids are compounds characterized by a vacant 2p orbital. This electron deficiency determines a chemical instability resulting in the formation of tetrahedral boron adducts owing to the attack of nucleophile agents, such as water, hydroxide, alkoxide, or amines. At room temperature, stable cyclic esters with saccharides are also formed through rapid and reversible reactions. Moreover, the aminoalkylboronic acids, boron analogues of common amino acids, as **BTZ** is, undergo a spontaneous 1,3-rearrangement to give the homologated amines, owing to the instability of free *α*-amino groups possessing hydrogen substituents. These compounds yield boric acids and alcohols by degradation and undergo oxidative reactions which easily destroy the C–B bond longer and weaker than the corresponding C–C bond. Figure 3 illustrates this characteristic reactivity (reactions a and b), which is of some interest for BTZ chemical stability [9–11].

The aim of this study is to explore the chemical stability of a commercial **BTZ** sample (Velcade) in its pharmaceutical form (i.e., stocked as a sterile, lyophilized formulation with mannitol as a bulking agent, in a glass vial filled with nitrogen), after its reconstitution according to the commercial reported instructions and stored at 4°C.

## 2. Materials and Methods

BTZ is commercialized by Millennium Pharmaceuticals (Mass, USA) in the US and Janssen-Cilag in Europe under the trade name Velcade, and is administered as intravenous bolus. The vials are reconstituted with 3.5 mL of sterile NaCl 0.9% to produce 1 mg/mL of BTZ and 10 mg/mL of mannitol. The product information states that reconstituted BTZ is stable for 8 hours when stored at <25°C and protected from light, and for 3 hours in a syringe.

The samples of **BTZ** under investigation were reconstituted using a sterile NaCl 0.9% solution (in deuterated water D_2_O, Merck) to produce the suitable NMR samples, according to the commercial reported instructions.

Sample A was used for a direct NMR investigation without further treatment, while the tubes containing the samples B and C were filled with argon (B) and oxygen (C), respectively. All the experiments were performed in triplicate.

The solutions A, B, and C, kept in dark at 4°C, were subjected to ^1^H NMR investigation and tested during a week to record possible differences in the mixture composition. After one month, the previously examined samples A, B, and C, kept in dark at 4°C, were reanalyzed by ^1^H NMR spectroscopy.

### 2.1. NMR and HPLC Experiments

Nuclear magnetic resonance (NMR) spectra were recorded at 500 MHz for [1H] and 12 MHz for ^13^C on a Fourier Transform NMR Varian 500 Unity Inova spectrometer. Carbon multiplicity was evidenced by DEPT experiments. HPLC analysis was performed at room temperature (∼25°C) using a Shimadzu LC-6A pump equipped with Rheodyne 7215 injection valve 20-mL, and a Shimadzu SPD-6A spectrophotometric detector working at 280 nm; a Symmetry C18 Waters column was employed. The mobile phase consisted of 40% (v/v) acetonitrile and 60% (v/v) 30 mM KH_2_PO_4_H_3_PO_4_ (pH 3.0). The product was eluted at 3.9 minutes with a flow rate of 1 mL/min.

## 3. Results and Discussion

Solutions A, B, and C, kept in the dark at 4°C, tested at intervals for a week by HPLC, did not differ between the initial and the final stage (Figure 6(a)). After one month, a small amount of two products eluted at 1 and 5 minutes was recorded (Figure 6(b)). According to this evidence, no change was observed in the NMR samples under the same conditions. After one month at 4°C, NMR reanalysis of the same samples A, B, and C showed that A and B were unchanged, whereas some modifications had taken place in the spectrum of solution C.

Particularly, the proton ($\underline{\text{H}}$, Figure 1) multiplet signal at 3.31 *δ* attributed to the hydrogen on the carbon linked to boron decreased in intensity by about 5%.

To investigate whether the origin of this difference was due to the presence of oxidation decomposition products, an air flow was gently bubbled, for six hours, through a **BTZ** sample reconstituted according to the commercial instructions and kept at 4°C.

**Figure 4:**
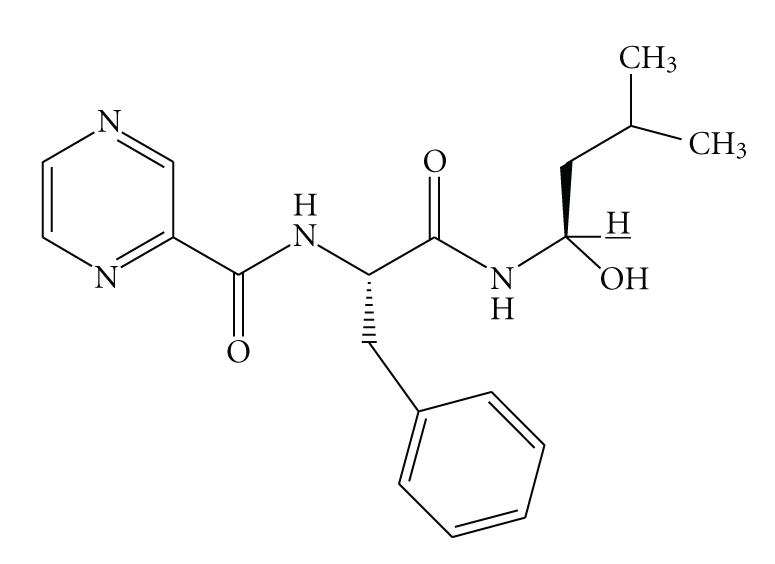
N-(1-(1-hydroxy-3-methylbutylamino)-1-oxo-3- phenylpropan-2-yl) pyrazine-2-carboxamide (**BTZ1**).

**Figure 5:**
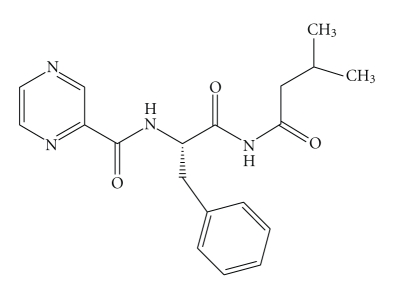
(S)-N-(1-(3-methylbutanamido)-1-oxo-3- phenylpropan-2-yl)pyrazine-2-carboxamide (**BTZ2**).

**Figure 6:**
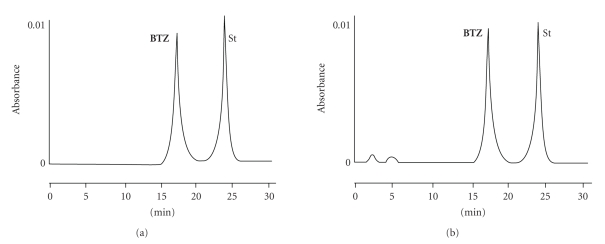
HPLC analysis of bortezomib solutions A, B, and C kept in dark at 4°C for a week. (a) No degradation product was observed. (b) After a month, a small amount of two new products, eluted at 1 and 5 minutes, respectively, was recorded.

The mixture was extracted with chloroform and analyzed chromatographically by HPLC. Together with the main **BTZ**, two new products, **BTZ1** and **BTZ2** (resp., ∼5% and 1% of starting **BTZ**), were recovered and investigated by NMR spectroscopy (see Figures 4  and 5). **BTZ1** showed an ^1^H NMR spectrum (CDCl_3_) with signals at *δ* 9.38 (1H, d; J = 1.5 Hz), 8.73 (1H, d; J = 2.6 Hz), 8.48 (1H, m), 8.28 (1H, bs), 8.02 (1H, bs), 7.26 (5H, m), 5.36 (1H, dd; J = 7, 5.5 Hz), 4.70 (1H, dd; J = 5.1, 9.0 Hz), 3.20 (1H, dd; J = 14, 5.1), 3.02 (1H, dd; J = 14, 9.1), 1.71 (1H, dd; J = 7, 14.4), 1.66 (1H, dd; J = 5.5, 14.4), 1.52 (1H, m), and 0.98 (6H, d). The ^13^C NMR spectrum showed signals at *δ* 171.3, 164.7, 148.5, 145.6, 144.7, 137.6, 135.9, 130.7 (2C), 129.1, 127.5 (2C), 74.4, 54.2, 43.6, 38, 24.1, and 22.4 (2C). The Mass spectra of **BTZ1** obtained from MALDI spectra recorded a peak at 339 m/e corresponding to the more stable protonated-dehydrated ion, (M + H − H_2_O, 100 %).

On the basis of the chemical and spectroscopic properties [12–16], the product **BTZ1** was identified as the N-(1-(1-hydroxy-3-methylbutylamino)-1-oxo-3-phenylpropan-2-yl) pyrazine-2-carboxamide. The decreasing intensity (about 5% in a month) of the signal at 3.31 *δ* present in the unchanged **BTZ** ($\underline{\text{H}}$ in Figure 1) recorded into the ^1^H NMR spectrum represents a clear indication that a chemical changing had happened at the carbon holding the hydrogen corresponding to this signal.

Moreover, the reproducibility of the ^1^H NMR spectra of samples A and B in time, clearly in contrast with the degradation of sample C, suggests that the oxygen bubbled into the C sample tube plays a determining role in the **BTZ** stability. According to the chemical behavior of boronic acid reported in literature, **BTZ** is sensitive to the oxidative effect of oxygen present in air and undergoes oxidative deboronation to **BTZ1**. 

Small amount (1% of starting **BTZ**) of another degradation product **BTZ2**, detected in the sample C, was recovered and investigated by NMR spectroscopy. **BTZ2** showed an ^1^H NMR spectrum (CDCl_3_) with signals at *δ* 9.38 (1H, d; J = 1.5 Hz), 8.73 (1H, d; J = 2.6 Hz), 8.67 (1H, bs), 8.48 (1H, m), 8.02 (1H, bs), 7.28 (5H, m), $\underline{4.91}$ ($\underline{1\text{H}}$, dd; J = 5.1, 9.1 Hz), 3.18 (1H, dd; J = 14.0, 5.1), 3.12 (1H, dd; J = 14.0, 9.1), 2.09 (2H, m), 1.72 (1H, m), and 1.02 (6H, d). The ^13^C NMR spectrum showed signals at *δ* 172.1, 171.3, 164.7, 148.5, 145.6, 144.7, 137.6, 135.9, 131.7 (2C), 129.3, 127.8 (2C), 55.1, 46.3, 38, 25.1, and 22.5 (2C).

The Mass spectra of **BTZ2** obtained from ES and MALDI spectra recorded a peak at 335 m/e corresponding to the protonated (M + H + 100%). Main characteristics of **BTZ**, **BTZ1**, and **BTZ2** are summarized in Table 1.

On the basis of the reported data, the structure of N-(1-(3-methylbutanamido)-1-oxo-3-phenylpropan-2-yl)pyrazine-2-carboxamide was attributed to **BTZ2** [12–16].

Our results suggest that even after reconstitution with saline, BTZ solutions are stable for at least one week in the dark at 4°C, and that very small amounts of BTZ derivatives are detectable in vials stored in the presence of oxygen after one month only. Thus, residual amounts of the drug in vials used for therapeutic purposes can be stored and reutilized within a few weeks, on the same patient or on different ones, without detectable loss of potency. These results may have interesting implications, both for patient management and in terms of cost effectiveness, particularly for centers treating small numbers of patients, considering the high cost of BTZ therapy.

## 4. Conclusions

The BTZ compound present in a reconstituted sample of Velcade kept at 4°C in dark resulted stable for a week, whereas it underwent oxidative transformation in presence of high air (oxygen) concentration after a month. The present result is not unexpected given the widely reported lability of boronic acids toward the oxidative deboronation.

## Figures and Tables

**Table 1:** Molecular weight, mass peak, and elemental analysis of **BTZ, BTZ1**, and **BTZ2**.

Comp.			Elemental analysis					
	MW	Mass peak m/z	C		H		N	
			calcd	found	calcd	found	calcd	found
**BTZ**	C_19_H_25_BN_4_O_4_	[M + H − H_2_O]^+^	59.39	59.48	6.56	6.58	14.58	14.61
	384	367						

**BTZ1**	C_19_H_24_N_4_O_3_	[M + H − H_2_O]^+^	64.03	64.12	6.79	6.81	15.72	15.70
	356	339						

**BTZ2 **	C_19_H_22_N_4_O_3_	[M+H]^+^	64.39	64.32	6.26	6.30	15.81	15.78
	354	355						
